# Tribological Performance of Bronze Engineering Materials with Environmentally Friendly Lubricants Under Starved Lubrication Conditions

**DOI:** 10.3390/ma18143283

**Published:** 2025-07-11

**Authors:** Marcin Kowalski, Kasper Górny, Szymon Bernat, Arkadiusz Stachowiak, Jacek Wernik, Wiesław Zwierzycki

**Affiliations:** 1 Faculty of Civil Engineering, Mechanics and Petrochemistry, Warsaw University of Technology Branch in Płock, Łukasiewicza 17, 09-400 Płock, Poland; marcin.kowalski2@pw.edu.pl (M.K.); jacek.wernik@pw.edu.pl (J.W.); 2 Institute of Machines and Motor Vehicles, Poznan University of Technology, Piotrowo 3, 60-965 Poznań, Poland; arkadiusz.stachowiak@put.poznan.pl (A.S.); wieslaw.zwierzycki@put.poznan.pl (W.Z.); 3 SINTEF Industry, Materials and Nanotechnology, Richard Birkelands vei 2 B, 7034 Trondheim, Norway; szymon.bernat@sintef.no

**Keywords:** friction coefficient, eco-friendly lubricants, starved lubrication

## Abstract

This article demonstrated that environmentally friendly lubricants—glycerol–water-based oil (GWB) and rapeseed oil-based oil (RSB)—would provide comparable conditions (wear of node components, friction resistance) in a friction node as a commercial semi-synthetic gear oil (REF). Wear tests were performed on a block-on-ring model friction node stand using GBZ12 (CuSn12), BA1032 (CuAl10Fe3Mn2), and BA1054 (CuAl10Ni5Fe4) bronze samples. Glycerol–water-based oil (GWB) significantly reduced the wear of the samples by several times, compared to semi-synthetic oil (REF) and rapeseed oil-based oil (RSB). The (GWB) oil also provided a stable friction coefficient value at the lowest level of 0.05–0.06. The main disadvantage of the (RSB) oil was the temporary fluctuation of the friction coefficient value (increase above 0.1), which indicated the lack of stability of the boundary layer. The results highlight the potential of (GWB) oil in reducing wear and stabilizing friction under extreme conditions, supporting the shift toward sustainable lubricants in industrial applications.

## 1. Introduction

Starved lubrication is considered to be a situation in which a small amount of lubricant is present in the friction node. This amount should enable the formation of a permanent boundary layer. Starved lubrication is often the way to lubricate machine nodes. When starved lubrication is intentionally forced, a distinction is made between drop lubrication, oil mist lubrication, and oil–air lubrication [1]. In general, it is noted in literature that starved lubrication reduces the thickness and extent of the oil film and raises the temperature in the friction node area [2]. It is also worth noting that starved lubrication, compared to full lubrication, carries the risk of greater damage [3].

In the case of small amounts of lubricant in the inlet contact of machine parts that form friction nodes with a concentrated point or linear contacts, for example, balls with a bearing raceway or a journal with a connecting rod bearing, situations may arise where the viscosity of the lubricant and velocity result in insufficient lubricant replenishment, conditions of starved lubrication, or no lubrication occurring locally [4]. Such conditions are often obtained for tribological model studies by placing one drop of lubricant on one of the samples that form a model friction node [5,6]. This amount is supposed to enable the formation of a boundary layer.

The actual components of sliding friction nodes, such as shafts, bearings, and bushings, in which the contact of the cooperating machine parts is circumferential, are well reflected by the block-on-ring model friction node [7,8]. It is worth noting that block-on-ring model friction nodes initially show concentrated linear contact, which, as the degree of wear increases, transitions to surface contact. Importantly, a moving roller in a block where a certain degree of wear is already present causes an uneven distribution of pressures across the cross-section of the block. Pressures in the critical zone, i.e., at the entrance and exit of the rotating roller, can be up to ten times higher than in the zone of normal pressures, i.e., at the bottom of the wear track. It is also noted in literature that key wear mechanisms can occur at the edges of the block wear track in the critical zone. The bottom of the wear track was identified as the normal wear zone, where lower-intensity wear mechanisms occur [8].

In tribological studies using the block-on-ring friction node, the wear track widths and friction coefficient values are used for comparison purposes, and SEM (scanning electron microscope) images of block-shaped samples are used to identify the wear mechanism [9]. In some studies, in addition to the wear track width, mass loss is analyzed, or wear volume is estimated using direct geometric measurements of wear tracks [8,10,11,12].

Lubricants also play an important role in machining, as they reduce the friction between the workpiece and the cutting tool, thus increasing the efficiency of the machining process and improving the durability of tools [13]. In order to achieve smooth operation, highest productivity, and efficiency, lubrication is a fundamental need for machines and equipment [14]. The demand for lubricants has increased tremendously due to the rapid growth of industrialization, which, in turn, increased the use of advanced machinery and equipment [15], and this continues to the present day.

Conventional lubricants and materials used in tribology are mainly derived from petroleum resources. Considering the environmental concerns and the increasing scarcity of resources, it is necessary to search for alternatives that can replace current lubricants in a sustainable manner [16,17]. The use of environmentally acceptable lubricants (EALs) is increasingly expected in industrial applications. However, their potential is still being explored worldwide in various applications. The results show that biodegradable and renewable resource-based lubricants are a promising alternative to conventional, traditional petroleum-based lubricants. Green lubricants, especially those derived from natural oils, have shown better tribological properties compared to conventional lubricants [18]. The development of biodegradable and renewable lubricants is crucial to minimize industrial pollution and meet stringent environmental regulations to reduce impacts on soil and water [19]. Bio-lubricants, produced from renewable resources, offer economic and environmental benefits, compared to mineral oil-based lubricants [20]. Environmentally acceptable lubricants are great alternatives to conventional lubricants because they are not only less toxic, more biodegradable, and renewable but also have exceptional lubricating properties, such as high lubricity, high viscosity index, high flash point, and low volatility [21,22,23,24].

Rapeseed oil has emerged as an environmentally friendly lubricant, and recent studies have shown its potential in tribological applications. Rapeseed oil shows promising physical and tribological properties, which make it a viable alternative to mineral lubricants [25,26,27,28,29]. Some studies indicate that the addition of nanoparticles, such as graphene nanoplatelets, CuO, TiO_2_, hexagonal boron nitride, and stearic acid, can significantly improve the performance of rapeseed oil-based lubricants by improving the viscosity index and enhancing tribological properties, including reducing friction coefficients and, consequently, reducing the wear volume [29,30,31,32]. For example, hybrid blends containing multiple nano-additives have shown up to a 20% reduction in friction and an 84% reduction in wear volume, compared to conventional lubricants [27]. Stearic acid as an additive has been shown to improve the anti-wear properties of rapeseed oil under boundary lubrication conditions at different temperatures [32]. The use of rapeseed biodiesel was associated with reduced friction and wear, with a noticeable 16% reduction in wear, compared to diesel fuel [33]. Kandeva et al. [34] showed that metal-containing additives in rapeseed oil can reduce the friction coefficient, operating temperature, and the degree of wear in steel–bronze tribological systems. In summary, rapeseed oil is a promising green lubricant, especially when modified with suitable additives, and is a viable alternative to traditional lubricants in various industrial applications as a way to reduce wear on machinery.

In addition to rapeseed oil, water-based lubricants (WBL) containing glycols are also a promising, environmentally friendly alternative to traditional oils in various applications. Water-based glycol lubricants significantly improve the friction and wear characteristics of various materials in industrial applications. In studies involving the AISI 52,100 bearing steel, these lubricants showed unique rheological properties, with viscosity-temperature relationships similar to conventional oils, but with lower pressure–viscosity ratios. WBLs generally exhibited lower friction coefficients compared to conventional oils, with polyethylene glycol (PEG) showing the lowest value of around 0.1 [35]. In addition, metal-doped carbon dots, especially the zinc-doped variants, showed a significant reduction in friction (62.5%) and wear (81.8%) when added to water-based lubricants [36]. Together, these results suggest that glycol–water-based lubricants, especially when enriched with specific additives, can provide excellent tribological properties, making them suitable for environmentally friendly industrial applications. In addition, the use of ionic liquids as additives to aqueous glycol lubricants has shown lower friction and wear coefficients compared to base greases, although they may not be superior to traditional organic friction modifiers [37]. Different metals show different reactions to aqueous glycol lubricants, influenced by their tribological and corrosion properties. Studies indicate that the introduction of ionic liquids into water–glycol systems improves lubrication and corrosion resistance, particularly by forming a protective adsorbed layer on metal surfaces, which improves the load-bearing capacity and reduces friction [38].

The interaction between different types of metals and environmentally friendly lubricants, such as rapeseed oil and water-based glycol lubricants, is complex and requires further research to optimize performance across different applications.

The tribological properties of CuSn12, a copper–tin alloy, are crucial to its performance in a variety of applications, particularly in self-lubricating bearings and filters. Research indicates that CuSn12 exhibits favorable wear and friction resistance, which is influenced by its microstructure and hardness. Studies show that alloy wear resistance correlates positively with hardness, suggesting that harder materials perform better under tribological stresses [39]. In addition, the formation of tribological layers during sliding contacts increases wear resistance, as these layers can lead to lower friction levels and increased durability [40]. The combination of these factors makes CuSn12 suitable for demanding environments, such as aviation and transportation, where high performance and reliability are paramount [41].

CuAl10Ni5Fe4 bronze shows improved wear resistance after short-term corrosive exposure, with a slight decrease in the friction coefficient after long-term corrosion [42]. Heat treatment significantly affects its tribological properties, with water quenching followed by aging at 300 °C showing the best results [43].

In general, the tribological properties of bronzes reveal significant insights into their wear resistance and friction characteristics. These bronzes show promising tribological properties suitable for applications in demanding environments, with specific compositions and reinforcements playing a key role in their performance. The main objective of this paper was to verify whether eco-friendly lubricants for heavy-duty machinery would provide comparable conditions (wear-of-node components, friction resistance) in a friction node as a commercial semi-synthetic gear oil (REF). Essentially, a glycerol–water-based prototype oil (GWB) developed for use on ships in contact with seawater was used for the tests in the study. The innovative nature of this oil was due to the fact that its composition met the environmental requirements for lubricants used in the marine environment. In addition, tests were conducted for another environmentally friendly oil based on rapeseed oil (RSB). This alternative lubricant could be used outside of a marine environment, in many industrial applications, where petroleum oils can be replaced (e.g., in hydraulic systems of agricultural machinery or as cutting fluids in machining) [44]. Nonetheless, these bio-lubricants present a viable option for applications with ecological risks or potential lubricant leakage.

Bio-lubricants derived from renewable sources offer significant environmental advantages over conventional petroleum-based lubricants, including biodegradability and reduced toxicity. These eco-friendly alternatives demonstrate superior lubricity, wear resistance, and viscosity index, compared to mineral oils. However, bio-lubricants also present disadvantages, such as lower thermo-oxidative stability, higher pour points due to moisture content, high heterogeneity, and increased production costs. For that reason, these lubricants require further research. The improvements by chemical modifications and additives can enhance their performance. All modifications should address remaining challenges to further improve the applicability and adoption of bio-lubricants in industrial applications to reduce dependence on petroleum resources [16,45,46]. In addition to modifying the lubricant compositions, it is also important to test other materials used in the production of industrial machines and equipment.

The tribological properties of the oils were tested on a model block-on-ring friction node. Assessments were made for extreme operating conditions—starved lubrication and high load. The friction coefficient value was recorded during the tests, and the wear of the sample was estimated afterward. The evaluation was performed by comparing the test results obtained in the friction node with environmentally friendly oils (GWB) and (RSB) with the results gained in the node with semi-synthetic oil (REF). Tests of each oil were provided for three sample materials. The tested bronzes were: GBZ12, BA1032, and BA1054. The variation of the sample material was to identify the possible influence of the material on the lubricating properties of the oil in the tested friction node.

According to the knowledge of the authors of this article, there are no publications on tribological studies of sliding friction nodes under starved lubrication conditions for the construction materials and oils considered in this article. The research results presented in the following sections of the article may provide an interesting supplement to tribological knowledge in this area. The presented studies used the previous experimental experience (and specialized stand) of the authors of the article in assessing the lubricating properties of refrigeration compressor oils under starved lubrication conditions [6].

## 2. Materials and Methods

In this study, the tribological tests under starved lubrication conditions were based on materials that are used in many industrial applications. Three types of bronzes were tested: GBZ12 (CuSn12), BA1032 (CuAl10Fe3Mn2), and BA1054 (CuAl10Ni5Fe4).

Tin bronze GBZ12 (CuSn12) has good wear and good corrosion resistance. It is used for machine parts requiring self-lubricating, anti-abrasive properties, e.g., for poorly lubricated and corroded bearings, machine parts, gears, and worm wheels. The hardness of this bronze is min. 90 HB, while its tensile strength is not less than 300 MPa. Table 1 shows the chemical composition of the GBZ12 (CuSn12) bronze.

BA1032 (CuAl10Fe3Mn2) bronze is an aluminum–iron–manganese bronze that is very hard and offers high resistance to corrosion and variable impact loads. In addition, it is resistant to static loads, abrasion, and elevated temperatures. It is used for highly stressed engine parts, as well as machine parts that are subjected to abrasion under high mechanical loads and exposed to corrosion. It is used in the communications, aerospace, marine, and chemical industries. The hardness of this bronze is 110–120 HB, while its tensile strength is 500–550 MPa. Table 2 shows the chemical composition of the BA1032 (CuAl10Fe3Mn2) bronze.

Bronze BA1054 (CuAl10Ni5Fe4) is one of the hardest bronze grades. It has very good strength properties, also at elevated temperatures, as well as good corrosion resistance, especially in acidic solutions. It is characterized by high resistance to variable loads and abrasion. It is used, for example, for sieve bottoms of heat exchangers, shafts, screws, components of hydraulic equipment, valve seats, and gears. The hardness of this bronze is 175–183 HB, while its tensile strength is min. 700 MPa. Table 3 shows the chemical composition of the BA1054 (CuAl10Ni5Fe4) bronze.

The surfaces of the samples were shaped by grinding and polishing. Profilometric measurements indicated that the roughness parameters Ra were around 0.18 µm for bronze GBZ12, 0.23 µm for bronze BA1032, and 0.31 µm for bronze BA1054.

The semi-synthetic gear oil Hipospec GL-5 75A/90 (REF) (Specol, Chorzów, Poland) was used as a reference for the tests in this study. The gear oil is widely used in industry, and it is designed for the lubrication of gears, especially hypoid gears of cars, trucks, buses, and other machinery and equipment operating under very difficult conditions. The research in this article provides a comparative basis of the reference oil with two oils developed with environmentally friendly ingredients. The glycerol–water-based oil (GWB) was obtained by mixing 95% glycerol, 4.9% water, and 0.1% wt. dodecanoic acid. The 2-dimethylaminoethanol was added to the fully formulated oil to increase the pH value (from 6.5 up to 9) and the degree of corrosion protection. The composition of the oil indicated its polarity. However, the formulation of the second oil, rapeseed oil-based oil (RSB), was based on non-polar rapeseed oil. The same friction modifier, namely dodecanoic acid, was added to the based oil, as in the case of the glycerol–water-based oil, in an amount of 0.1 wt.%.

**Figure 1:**
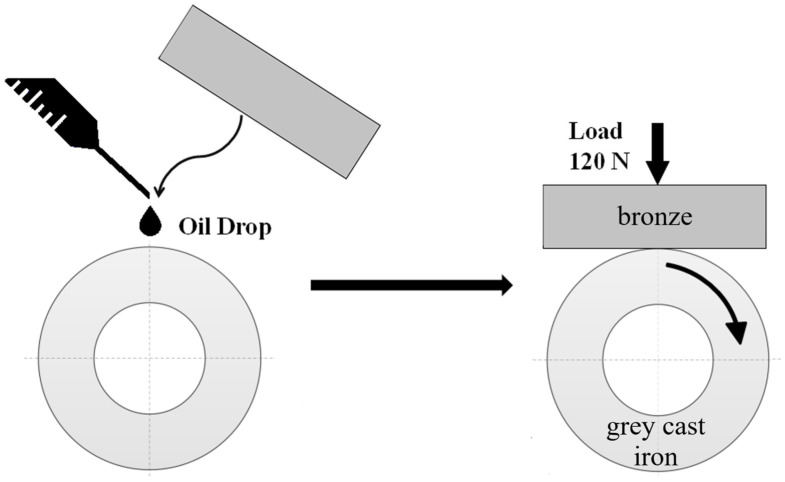
Block-on-ring node used for lubricity evaluation under starved lubrication conditions.

To assess the lubricating properties under starved lubrication conditions, a test stand was used, which was based on the wear of a model block-on-ring friction node (Figure 1). The tested samples made of bronzes were implemented in the friction nodes in the form of a block, where the ring (counter-sample) was manufactured with gray cast iron EN-GJL-250. Gray cast iron EN-GJL-250 is characterized by the following properties: density of about 6.8–7.3 g/cm^3^, tensile strength of about 250 MPa, yield strength of about 140 MPa, and hardness of about 260 HB.

In the proposed test method, the sample wear process occurred under conditions that allowed for a clear material loss in a relatively short time and reflected the actual operating conditions of friction nodes under starved lubrication conditions.

Regarding the test procedure, before each test, the samples had to be cleaned in an ultrasonic cleaner, using acetone, for 15 min. Subsequently, they were mounted in the test chamber. The appropriate amount of lubricant had to be applied before the wear test. The test parameters for the lubricating properties of the oils under starved lubrication and no lubrication conditions are presented in Table 4.

**Figure 2:**
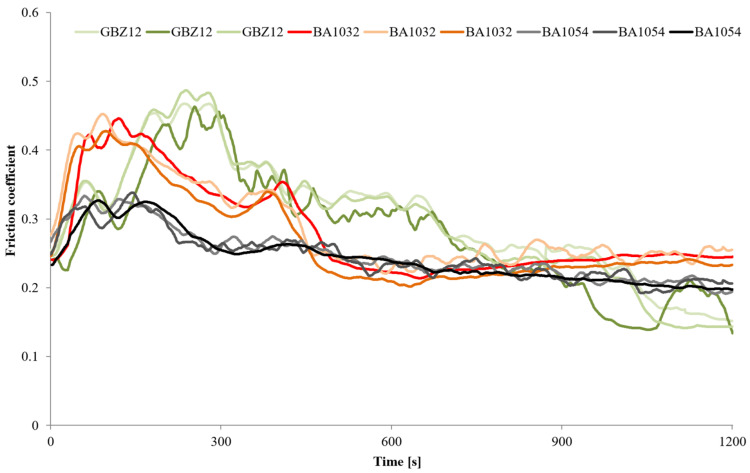
Friction coefficient values for the three materials without lubrication and with a load of 120 N.

**Figure 3:**
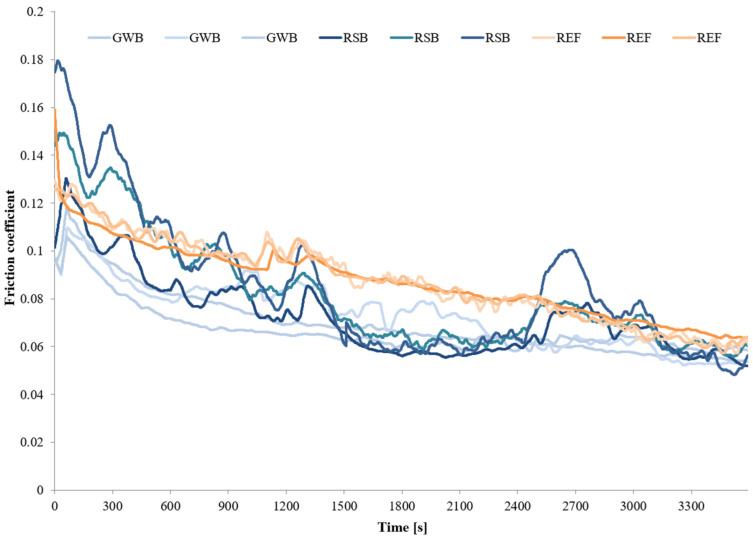
Friction coefficient values for GBZ12 under starved lubrication conditions with all the tested lubricants at a constant load of 120 N.

**Figure 4:**
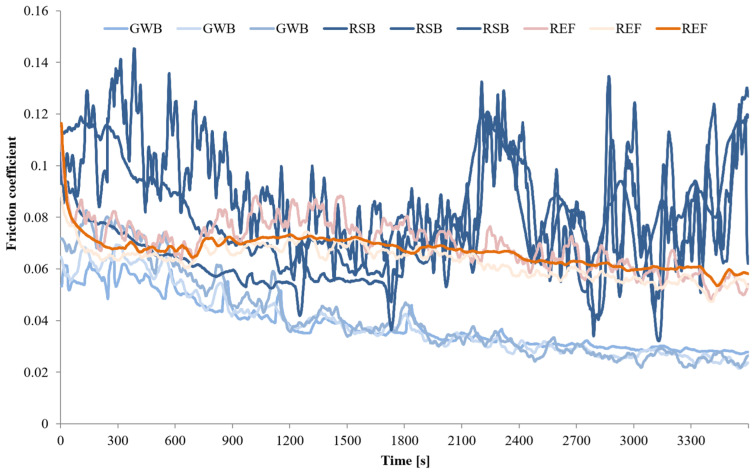
Friction coefficient values for BA1032 under starved lubrication conditions with all the tested lubricants at a constant load of 120 N.

**Figure 5:**
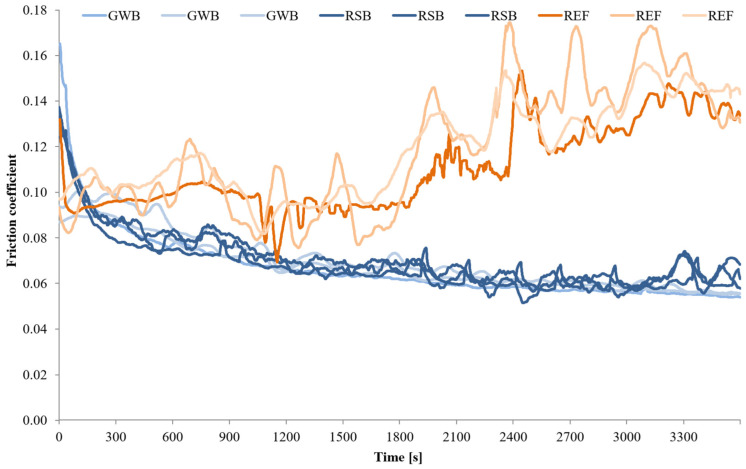
Friction coefficient values for BA1054 under starved lubrication conditions with all the tested lubricants at a constant load of 120 N.

**Figure 6:**
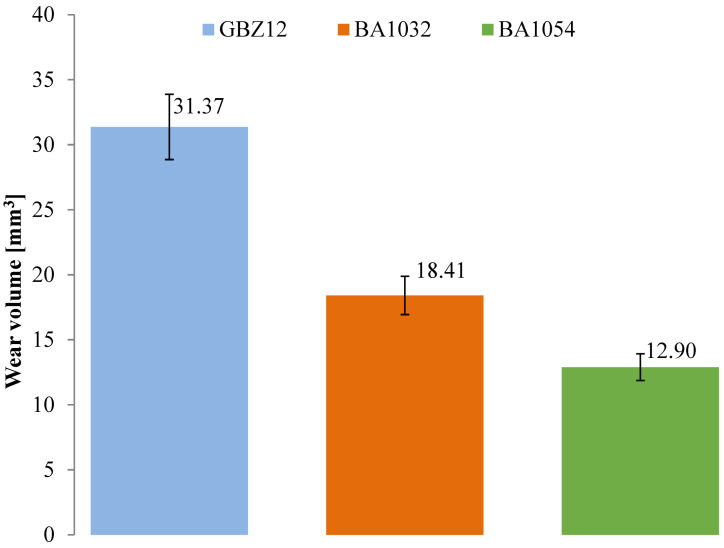
The average volumetric wear of the samples after tests without lubrication for the different materials.

**Figure 7:**
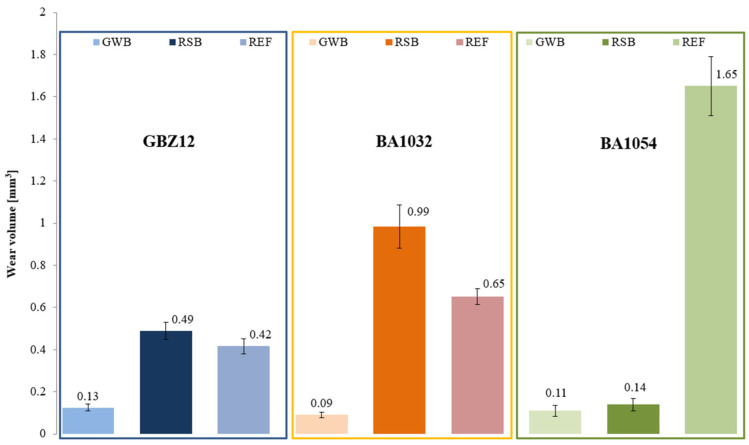
The average volumetric wear of the samples after tests under starved lubrication conditions for the different materials.

The wear tests for each material (GBZ12, BA1032, BA1054) series with no lubricant and with a small amount of suitable lubricant (GWB, RSB, REF) were performed in the friction node according to the guidelines in Table 4. Three wear tests were performed for each of the test combinations. The measurements were conducted at room temperature (20 °C). Figure 2, Figure 3, Figure 4 and Figure 5 present the selected friction coefficient curves, while Table 5 and Table 6 present average friction coefficient values for individual tests in the series for the tested bronzes without and with oil. Table 7 and Figure 6 and Figure 7 show the volumetric wear values of the investigated samples. Figure 6 and Figure 7 exhibit the mean value for the test series and a measure of the scatter (standard deviation).

## 3. Results and Discussion

The volumetric wear of the sample was determined by the following Formula (1):

(1) $$V=0.5sr^{2}\left\{2arcsin\left(\frac{x}{2r}\right)-sin\left[2arcsin\left(\frac{x}{2r}\right)\right]\right\}$$

where: *V*—volumetric wear of the sample [mm^3^], *s*—width of the sample (block) [mm], *r*—radius of the counter sample (roller) [mm], and *x*—width of the wear track of the sample [mm]. (The measurement of the width of the wear track for the sample (block) was made using a Brinell magnifier, with an accuracy of 0.1 mm.)

The method used by the authors of this paper was intended to evaluate the comparative lubricating properties of lubricants. Among the tested oils, the one for which the effects (volumetric wear of a block-shaped sample in a ring association) caused by the same force were smaller had better lubricating properties.

Figure 2 shows the friction coefficient values for the three materials used in the study without lubrication at a constant load of 120 N (three tests for each material sample). The duration of each test was 20 min. Table 5 presents the average friction coefficient values for each test (values in columns: no. 1, 2, 3). Averaging was performed for the last 300 s of the test, when stabilization occurred in most cases. Based on these three values, an average value characterizing the series of tests for a given material was also determined. The friction coefficient average values in the final phase of the test were 0.186 for bronze GBZ12, 0.243 for bronze BA1032, and 0.207 for bronze BA1054 (Table 5). In general, the average friction coefficient values for the tested materials did not differ significantly.

The results characterized the frictional interactions of the bronzes tested in pairs with the counter-sample material under dry friction conditions and served as a comparative evaluation of the bronzes tested. The results provide a reference for further research under starved lubrication conditions.

The friction coefficient values for a series of tests on the individual materials (GBZ12, BA1032, BA1054) with lubricants (GWB, RSB, REF) under starved lubrication conditions are shown in Figure 3, Figure 4 and Figure 5. The duration of each test was 60 min at a load of 120 N. Figure 3, Figure 4 and Figure 5 show all the friction coefficient variations (three tests in each test series of the material sample–oil-type pair). In addition, Table 6 shows the average friction coefficient values for each test. The averaged values were determined for the final part of the test (last 600 s), where relative stabilization occurred.

In the initial phase of the test, the curves showed more variation than in the final phase. In the initial phase of the test, the frictional interactions were intense (high pressure in the contact zone, intensive micro-cutting, and associated resistance to movement). At the same time, the random nature (nature of the friction process) of the frictional interactions can cause dynamic fluctuations in the friction coefficient. During the tribological test, the friction coefficient value decreased. This may be due to the fact that the size of the wear track increased with time, along with the nominal contact area of the sample and the counter-sample as well. These changes caused the unit pressure in the contact zone to decrease. Therefore, it was easier to create and maintain a boundary layer of lubricant that separated the cooperating elements of the friction node.

Figure 3 illustrates the changes in the friction coefficient in tests with GBZ12 bronze samples and all tested oils. The average values of the friction coefficient in Table 6 indicate that the resistance to movement in the friction node was similar for all the GBZ12 bronze tests with the three analyzed oils. In the final part of the test, the average friction coefficient values were: 0.057 for the glycerol–water-based oil (GWB), 0.060 for the rapeseed oil-based oil, and 0.064 for the semi-synthetic oil (REF). The variation of the friction coefficient in tests with the BA1032 bronze samples is shown in Figure 4. In the case of this material, the friction coefficient value stabilized at the end of the experiment at 0.027 for oil (GWB) and 0.056 for oil (REF). For oil (RSB), frequent and large fluctuations in the range 0.06–0.12 were visible. For the hardest bronze BA1054 (Figure 5), in the final part of the wear test, the coefficients of friction (average value) were 0.056 for oil (GWB), 0.062 for oil (RSB), and 0.146 for oil (REF). In the case of oil (GWB), the values stabilized, while sporadic fluctuations were noticeable for the other oils.

The friction coefficient values observed during the tests—under starved lubrication conditions—were mostly in the range of 0.06–0.10 (in the main, final part of the test). These values indicated that mixed friction was present in the friction node. Such interaction conditions may explain the temporary fluctuations in friction coefficient values, depending on the currently prevailing conditions in the contact zone (roughness and formation of wear particles). There was direct contact between the surfaces of the sample and the counter-sample (dry friction), or the surfaces were separated by a lubricant film (boundary and fluid friction). The fluctuation of the friction coefficient (rapid rise) might be mostly the result of the third body particle released and the breakdown of the lubricating film. The reduction of the friction coefficient for all the tests was due to the running, which resulted in surface alignment and, therefore, the reduction of overall maximum contact pressure.

The results shown in Figure 6 refer to the average volumetric wear of the samples after tests without lubrication for the different materials. In these series, no lubricant was added, and the duration of each test was 20 min.

In the case of GBZ12, the volumetric wear without lubrication was 31.37 mm^3^; for BA1032, it was 18.41 mm^3^; and for BA1054, it was 12.90 mm^3^. The BA1032 material showed a higher wear resistance, almost twice as high as the GBZ12 material. However, the wear resistance of BA1054 was about 30% better than that of BA1032 and about 2.5 times better than that of GBZ12.

Table 7 presents the volumetric wear of samples made of bronze materials after tests with the investigated lubricants under starved lubrication conditions, where the duration of each test was 60 min. The table shows the results of three tests in each series of tested material–oil pairs, the mean value for the series, and the standard deviation. In graphical form, the results are shown in Figure 7.

In the case of GBZ12/GWB, the volumetric wear was 0.13 mm^3^; for the GBZ12/RSB series, the wear was 0.49 mm^3^. However, for the GBZ12/REF series, it was 0.42 mm^3^. The results showed that lubrication properties were almost four times better with a small amount of lubricant in the friction node when using GWB oil as compared to RSB for the GBZ12 sample material. The lubricating properties of the REF oil were by approximately 230% worse than those of the GWB oil.

The volumetric wear was 0.09 mm^3^ for BA1032/GWB, 0.99 mm^3^ for BA1032/RSB, and 0.65 mm^3^ for the BA1032/REF series. The results showed that lubrication properties were almost eleven times better with the presence of a small amount of lubricant in the friction node when using GWB oil as compared to RSB for the BA1032 sample material. The lubricating properties of the REF oil were approximately 625% worse than those of the GWB oil.

For BA1054/GWB, the volumetric wear was 0.11 mm^3^; for BA1054/RSB, it was 0.14 mm^3^; and for BA1054/REF, it was 1.65 mm^3^. The research results demonstrated lubrication properties that were fifteen times better with the presence of a small amount of lubricant in the friction node when using GWB oil as compared to REF for the BA1054 sample material. The lubricating properties of the RSB oil were approximately 27% worse than those of the GWB oil.

**Figure 8:**
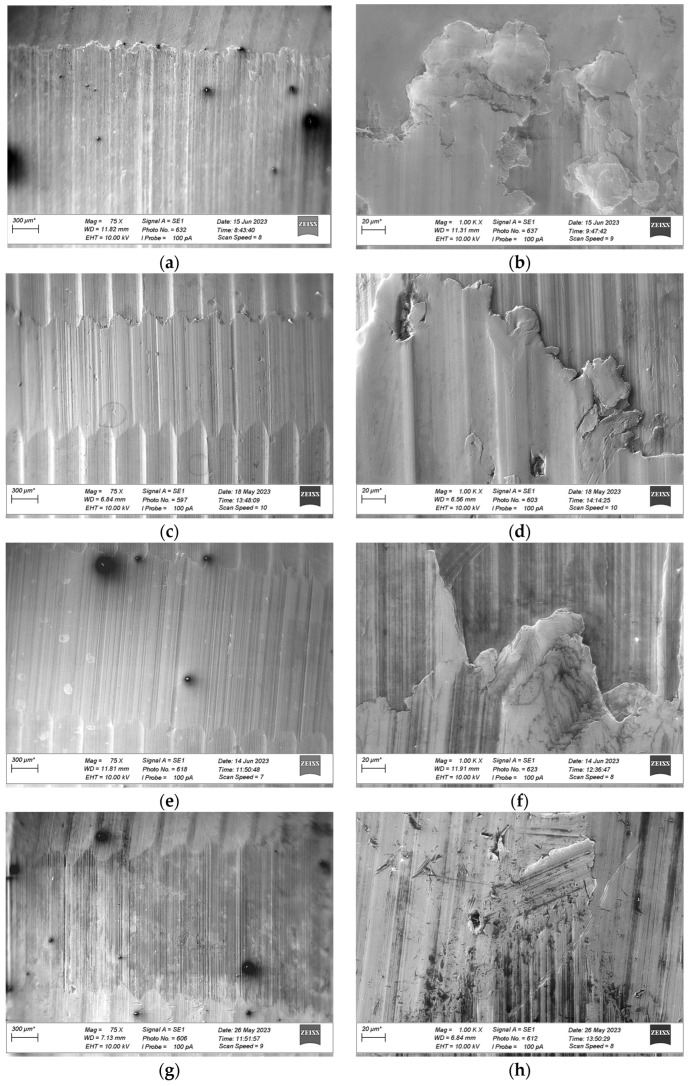
SEM images of the wear tracks formed on GBZ12 material during tribological tests under starved conditions with different types of lubricants and no lubricant: (**a**) no lubricant with a magnification of 75 times; (**b**) no lubricant with a magnification of 1000 times; (**c**) GWB with a magnification of 75 times; (**d**) GWB with a magnification of 1000 times; (**e**) RSB with a magnification of 75 times; (**f**) RSB with a magnification of 1000 times; (**g**) REF with a magnification of 75 times; (**h**) REF with a magnification of 1000 times (*—the length of the reference is individual for each drawing).

**Figure 9:**
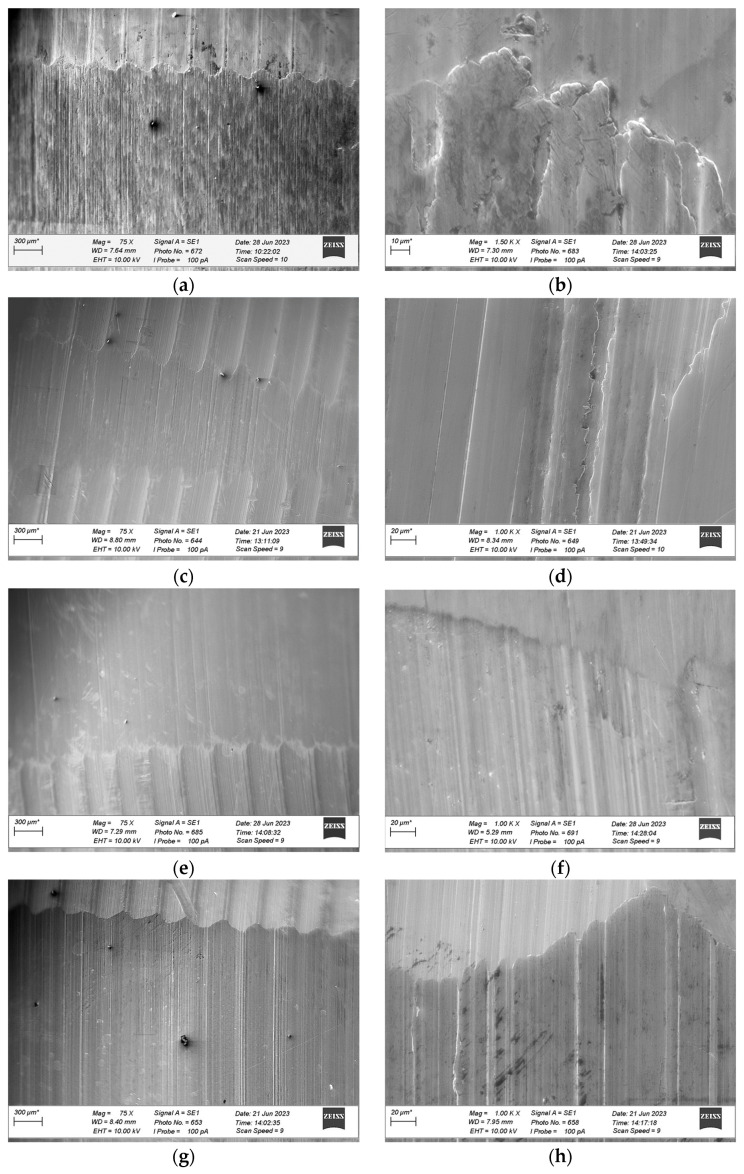
SEM images of the wear tracks formed on BA1032 material during tribological tests under starved conditions with different types of lubricants and no lubricant: (**a**) no lubricant with a magnification of 75 times; (**b**) no lubricant with a magnification of 1000 times; (**c**) GWB with a magnification of 75 times; (**d**) GWB with a magnification of 1000 times; (**e**) RSB with a magnification of 75 times; (**f**) RSB with a magnification of 1000 times; (**g**) REF with a magnification of 75 times; (**h**) REF with a magnification of 1000 times (*—the length of the reference is individual for each drawing).

**Figure 10:**
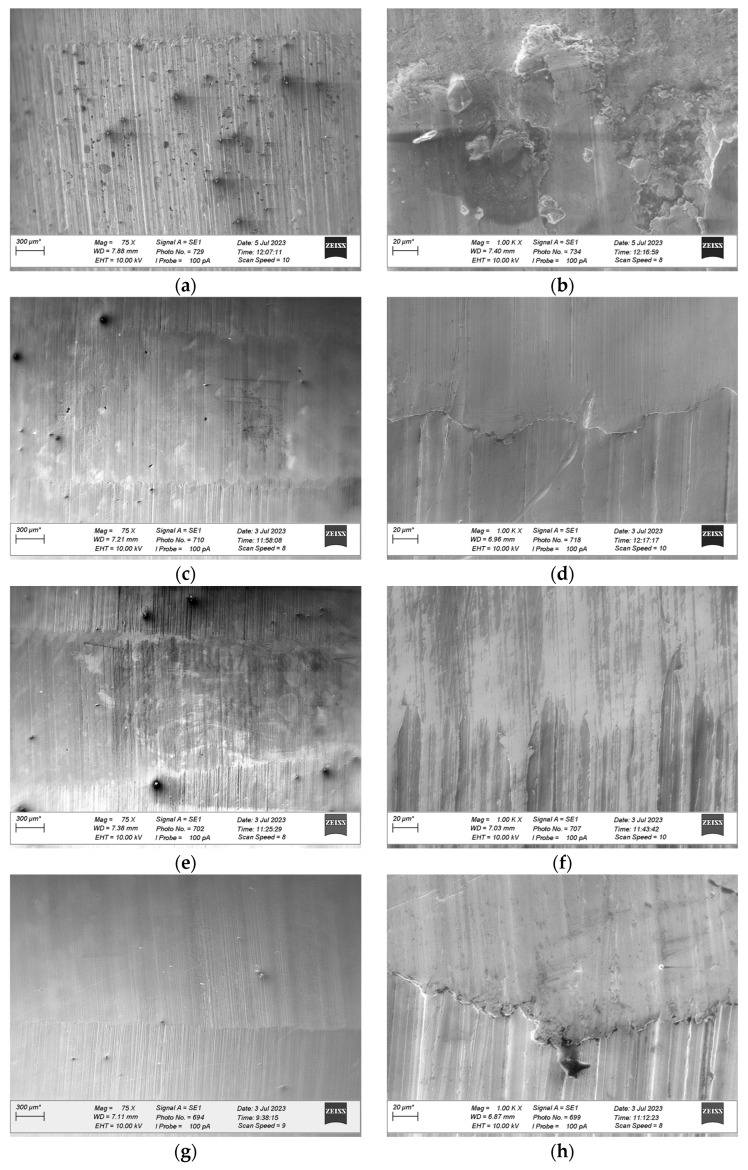
SEM images of the wear tracks formed on BA1054 material during tribological tests under starved conditions with different types of lubricants and no lubricant: (**a**) no lubricant with a magnification of 75 times; (**b**) no lubricant with a magnification of 1000 times; (**c**) GWB with a magnification of 75 times; (**d**) GWB with a magnification of 1000 times; (**e**) RSB with a magnification of 75 times; (**f**) RSB with a magnification of 1000 times; (**g**) REF with a magnification of 75 times; (**h**) REF with a magnification of 1000 times (*—the length of the reference is individual for each drawing).

After wear tests were completed, microscopic observations of the wear tracks were performed using a ZEISS EVO scanning electron microscope (ZEISS, Munich, Germany). The results are shown in Figure 8, Figure 9 and Figure 10. The left column of each figure (labels a, c, e, and g) contains images taken at a magnification of 75 times. The images illustrate the surface of the wear track and a section of the sample surface next to the track. The right column (labels b, d, f, and h) contains images taken at a magnification of 1000 times. The images illustrate the surface of the wear track in its central part. Based on microscopic observations of the surface of the wear track, the damage mechanisms were identified [49,50].

Figure 8a, Figure 9a and Figure 10a show the wear tracks of the tested materials after wear tests were performed under dry friction conditions (without lubricant). All wear track surfaces presented characteristics of abrasive wear—grooves consistent with the direction of rotation of the counter-sample were visible. The mentioned form of wear was the effect of the mechanical action of the surface roughness appearances of the cooperating elements due to their direct contact. This type of contact (dry friction) was indicated by friction coefficient values higher than 0.1 (Figure 2).

SEM images marked with symbols c, e, and g in Figure 8, Figure 9 and Figure 10 illustrate the wear tracks of the tested bronzes after wear tests in the presence of oils. The friction coefficient values observed during these tests (Figure 3, Figure 4 and Figure 5) indicated that mixed friction occurred in the contact area of the sample and counter-sample. (In the main, final part of the test, the friction coefficient was in the range of 0.03–0.10).

The tested oils were able to create a boundary layer on the cooperating surfaces of the sample and the counter-sample separating them. The formation of such a layer was particularly favored by the dodecanoic acid contained especially in the GWB oil. The model of the formation of a stable boundary layer by oil with the addition of dodecanoic acid was described in earlier publications by one of the co-authors of this study [49]. In the case of GWB oil, there were no major fluctuations in the friction coefficient, which may indicate the durability of the boundary layer. For the other oils, temporary increases in the friction coefficient value were visible. They may be the effect of the disruption (removal) of the boundary layer (or the formation of a wear particle). In such cases, direct contact with the cooperating surfaces may occur.

In the area of the wear tracks of the tested bronzes after wear tests in the presence of oils, forms of material removal characteristic of abrasive wear were visible. In the initial phase of the test and during the temporary disruption of the lubricant boundary layer, direct contact with the surface roughness appearances of the sample and counter-sample occurred. The effect of such interactions was parallel grooves (scratches) in the direction of rotation of the counter-sample. However, these forms were “milder” than in the case of dry friction (tests without oil). The mentioned grooves were fewer and narrower. The presence of the lubricant boundary layer resulted in the plastic deformation of the sample surface roughness appearances due to the contact rather than their mechanical separation (micro-cutting). This difference was observed in the case of the bronze with the lowest hardness, GBZ12, after tests in GWB and REF oils. In the case of a stable boundary layer generated by GWB oil, local plastic deformations were noticed at a magnification of (1000×) (Figure 8d). The plastic deformation was characterized by soft edges of smeared material removed from the contact point to the side. For REF oil (slightly higher values of the friction coefficient (Figure 3)), observations at the same magnification illustrated tracks of micro-cutting (Figure 8h). The presence of scratches and sharp edges indicated abrasive wear.

Of the bronze samples tested, the lowest hardness sample, GBZ12, showed a significant reduction in wear after lubrication with GWB and REF, in contrast to the unlubricated sample, where the degree of wear was the highest among the samples tested. This observation was particularly significant with GWB lubrication. In the case of a harder bronze material, BA1032, all lubricants significantly reduced the degree of wear, compared to the unlubricated samples. Again, GWB stood out for its better wear reduction, with significantly less plastic deformation and material flow in the brass, which can probably be attributed to the higher hardness of BA1032, compared to GBZ12. Importantly, the wear mechanisms appeared to be consistent across all lubricants for BA1032, despite the different wear rates. The only wear track that showed significant plastic flow in the bronze material was the unlubricated BA1032 sample, potentially due to a significant increase in contact temperature, leading to localized melting. The last material tested, BA1054, showed unique properties. In this case, tracks of brass smearing were particularly evident at the edges of the wear track lubricated with REF samples. This finding suggests elevated local temperatures (confirmed with high friction forces), possibly increasing the reactivity of the extreme pressure additives in the REF lubricant, leading to the removal of the sacrificial tribolayer, facilitating higher wear. The other GWB and RSB oils did not cause such effects in the form of wear. The low wear for the GWB and RSB lubrications of the BA1054 bronze was due to the resistance of this bronze to wear after the oil film broke, which was confirmed by tests without lubrication (Figure 6).

## 4. Conclusions

The main objective of this article was to verify whether eco-friendly lubricants for heavy-duty machinery would provide comparable conditions (wear of node components, friction resistance) in a friction node as a commercial semi-synthetic gear oil (REF). The glycerol–water-based oil (GWB) and the rapeseed oil-based oil (RSB) were tested against commercial gear oil (REF) on GBZ12 (CuSn12), BA1032 (CuAl10Fe3Mn2), and BA1054 (CuAl10Ni5Fe4) bronzes. The evaluation was exhibited for extreme operating conditions—starved lubrication and high load. The following conclusions were drawn from the test results:

- In tribological tests with glycerol–water-based oil (GWB) for each sample material, the lowest friction coefficient values were observed in the final phase of the test (Figure 3, Figure 4 and Figure 5). The friction coefficients were at the levels of 0.057 for the GBZ12 material, 0.027 for the BA1032 material, and 0.056 for the BA1054 material. These values indicated that mixed friction occurred in the tested node. Low and especially stable friction coefficient values distinguished that the oil (GWB) was able to create and maintain a durable boundary layer on the surfaces of the sample and counter-sample. This ability may result from the presence of additives in the oil in the form of dodecanoic acid.
  The durable boundary layer also ensured the lowest wear of the sample (for all tested bronzes) in tests with glycerol–water-based oil (GWB). The mentioned volumetric wears of the sample were 0.13 mm^3^ for GBZ12, 0.09 mm^3^ for BA1032, and 0.11 mm^3^ for BA1054 (Figure 7). For comparison, after tests with semi-synthetic oil (REF), the wears of the sample were 0.42 mm^3^ for GBZ12, 0.65 mm^3^ for BA1032, and 1.65 mm^3^ for BA1054 (Figure 7).
- In tribological tests with rapeseed oil-based oil (RSB), the average friction coefficient values in the final test phase were at the levels of 0.060 for the GBZ12 material, 0.083 for the BA1032 material, and 0.062 for the BA1054 material. These values are similar to the results of tests with water-based oil (GWB) and lower than in the case of tests with semi-synthetic oil (REF).
- The main drawback in the friction node lubricated with rapeseed oil-based oil (RSB) was the temporary fluctuation of the friction coefficient (generally an increase above 0.1). This was particularly visible in the case of tests with BA1032 bronze samples. Temporary increases in the friction coefficient may indicate an unstable boundary layer. In such conditions (dry friction, direct contact of the sample, and counter-samples), the wear of the samples in the node with oil (RSB) was higher than in the case of glycerol–water-based oil (GWB).
- The findings highlight the potential of (GWB) oil in reducing wear and stabilizing friction under extreme conditions, supporting the shift toward sustainable lubricants in industrial applications. Further optimization through using different materials for test samples and different lubricants with various concentrations of additives or their modifications, such as carboxylic acids with different chain lengths or amine groups, could enhance their effectiveness.

## Figures and Tables

**Table 1:** Chemical composition of the GBZ12 (CuSn12) [47].

Element	Cu	Sn	Zn	Pb	Al	Fe	Mn	Ni	Si	P
Weight, %	Rest	11.0–13.0	max 0.50	max 0.70	max 0.01	max 0.20	max 0.20	max 2.0	max 0.01	max 0.60

**Table 2:** Chemical composition of the BA1032 (CuAl10Fe3Mn2) [48].

Element	Cu	Sn	Zn	Pb	Al	Fe	Mn	Ni	Si
Weight, %	Rest	max 0.1	max 0.50	max 0.05	9.0–11.0	2.0–4.0	1.5–3.5	max 1.0	max 0.20

**Table 3:** Chemical composition of the BA1054 (CuAl10Ni5Fe4) [48].

Element	Cu	Sn	Zn	Pb	Al	Fe	Mn	Ni	Si
Weight, %	Rest	max 0.1	max 0.40	max 0.05	8.5–11.0	3.0–5.0	max 1.0	4.0–6.0	max 0.20

**Table 4:** Selected test parameters under starved lubrication conditions.

Parameter	Unit	Starved Lubrication	No Lubrication
Sliding speed	[m/s]	0.5	
Load of friction node	[N]	120	
Amount of lubricant	mg	30	0
Wear test time τt	[min]	60	20

**Table 5:** Average friction coefficient values for individual tests in the series for the tested bronzes without oil.

Block-Shaped Sample Material	Friction Coefficient [-]			
	No 1	No 2	No 3	Mean Value
GBZ12	0.173	0.184	0.201	0.186
BA1032	0.245	0.234	0.249	0.243
BA1054	0.205	0.208	0.207	0.207

**Table 6:** Average friction coefficient values for individual tests in the series for the tested bronzes with oils (material sample–oil pairs).

Block-Shaped Sample Material	Lubricant	Friction Coefficient [-]			
		No 1	No 2	No 3	Mean Value
GBZ12	GWB	0.056	0.055	0.060	0.057
	RSB	0.059	0.062	0.059	0.060
	REF	0.067	0.063	0.063	0.064
BA1032	GWB	0.029	0.026	0.025	0.027
	RSB	0.084	0.087	0.079	0.083
	REF	0.059	0.056	0.054	0.056
BA1054	GWB	0.055	0.056	0.058	0.056
	RSB	0.060	0.062	0.065	0.062
	REF	0.139	0.151	0.148	0.146

**Table 7:** Wear results of all tribological tests under starved lubrication conditions.

Block-Shaped Sample Material	Lubricant	Wear Volume [mm^3^]				Standard Deviation [mm^3^]
		No 1	No 2	No 3	Mean Value	
GBZ12	GWB	0.1395	0.1099	0.1395	0.1296	0.0171
	RSB	0.4646	0.4646	0.5343	0.4878	0.0402
	REF	0.4012	0.4012	0.4646	0.4223	0.0366
BA1032	GWB	0.0865	0.0865	0.1099	0.0943	0.0135
	RSB	0.9258	1.1041	0.9258	0.9852	0.1094
	REF	0.6308	0.6942	0.6308	0.6519	0.0366
BA1054	GWB	0.0865	0.1395	0.1099	0.1119	0.0266
	RSB	0.1690	0.1395	0.1099	0.1394	0.0295
	REF	1.7504	1.5704	1.8117	1.6508	0.1393

## Data Availability

The original contributions presented in the study are included in the article. Further inquiries can be directed to the corresponding author.

## References

[1] Stachowiak G., Batchelor A.W. (2013). Engineering Tribology.

[2] Bhutta M.U., Khan Z.A., Garland N. (2019). Wear Performance Analysis of Ni–Al_2_O_3_ Nanocomposite Coatings under Nonconventional Lubrication. Materials.

[3] Zhang Z., Shao F., Liang Y., Lin P., Tong X., Ren L. (2017). Wear Behavior of Medium Carbon Steel with Biomimetic Surface Under Starved Lubricated Conditions. J. Mater. Eng. Perform..

[4] Poll G., Li X., Bader N., Guo F. (2019). Starved Lubrication in Rolling Contacts-A Review. Bear. World J..

[5] Zhao C., Ying L.X., Li D.H., Wang D., Hu J.S. (2023). Influence of Chief Vein on Tribological Behavior of Vein-Bionic Textured Rolling Element Bearings Under StarvedLubrication. Tribol. Trans..

[6] Górny K., Stachowiak A., Tyczewski P., Zwierzycki W. (2022). Mixtures of Lubricants and Ecological Refrigerants under Starved Lubrication Conditions. Materials.

[7] Saba N., Jawaid M., Alothman O.Y., Paridah M.T. (2016). A review on dynamic mechanical properties of natural fibre reinforced polymer composites. Constr. Build. Mater..

[8] Awwad K.Y.E., Fallahnezhad K., Yousif B.F., Mostafa A., Alajarmeh O., Shalwan A., Zeng X. (2024). Finite element analysis and experimental validation of polymer–metal contacts in block-on-ring configuration. Friction.

[9] Wu H., Yin S., Du Y., Wang L., Wang H. (2020). An investigation on the lubrication effectiveness of MoS_2_ and BN layered materials as oil additives using block-on-ring tests. Tribol. Int..

[10] Akagaki T., Kuraoka Y., Takeo F., Furuya K., Kawabata M. (2017). Effects of PEEK’s surface roughness on seizure behaviors of PEEK/steel pairs under oil-lubricated sliding contacts. Mech. Eng. J..

[11] Du F., Li D., Sa X., Li C., Yu Y., Li C., Wang J., Wang W. (2022). Overview of Friction and Wear Performance of Sliding Bearings. Coatings.

[12] Zhang L., Chen Q., Yin Y., Song H., Tang J. (2024). Effects and optimization of bionic texture parameters on the tribological behavior of line contacts under starved lubrication conditions. Ind. Lubr. Tribol..

[13] Spikes H. (2015). Friction modifier additives. Tribol. Lett..

[14] Yelchuri V., Azmeera T., Karuna M.S.L. (2019). Metathesized castor oil acylated derivatives: Lubricants base stocks with low pour points and superior anti-wear properties. SN Appl. Sci..

[15] Zulkifli N.W.M., Kalam M.A., Masjuki H.H., Al Mahmud K.A.H., Yunus R. (2014). The effect of temperature on tribological properties of chemically modified bio-based lubricant. Tribol. Trans..

[16] Malik M.A.I., Kalam M.A., Mujtaba M.A., Almomani F. (2023). A Review of Recent Advances in the Synthesis of Environmentally Friendly, Sustainable, and Nontoxic Bio-lubricants: Recommendations for the Future Implementations. Environ. Technol. Innov..

[17] Negi P., Singh Y., Tiwari K. (2021). A Review on the Production and Characterization Methods of Bio-based Lubricants. Mater. Today: Proc..

[18] Reeves C.J., Menezes P.L., Davim J.P. (2016). Advancements in Eco-Friendly Lubricants for Tribological Applications: Past, Present, and Future. Ecotribology.

[19] Prema E., Sundar V.S., Lynch M., Sivaraman K., Venkatesan R. (2023). The need for eco-friendly green bio-lubricants to achieve united nations sustainable development goals: An analysis. Proceedings of the 14th International Conference on Materials Processing and Characterization.

[20] Shah R., Woydt M., Zhang S. (2021). The Economic and Environmental Significance of Sustainable Lubricants. Lubricants.

[21] Singh R.K., Singh A.K. (2014). Abilities of some compounds to stabilize mahwa oil from high temperature oxidative degradation for biolubricant applications. Waste Biomass-Valorization.

[22] Joseph P.V., Sharma D.K. (2010). Improvement of thermo-oxidative stability of non-edible vegetable oils of Indian origin for biodegradable lubricant application. Lubr. Sci..

[23] Jayadas N.H., Prabhakaran N.K., Ajithkumar G. (2007). Tribological evaluation of coconut oil as an environment-friendly lubricant. Tribol. Int..

[24] Krzan B., Vizintin J. (2003). Tribological properties of an environmentally adopted universal tractor transmission oil based on vegetable oil. Tribol. Int..

[25] Baskar S., Sriram G., Arumugam S. (2017). Tribological Analysis of a Hydrodynamic Journal Bearing under the Influence of Synthetic and Biolubricants. Tribol. Trans..

[26] Joseph P.V., Saxena D., Sharma D.K. (2007). Study of some non-edible vegetable oils of Indian origin for lubricant application. J. Synth. Lubr..

[27] Nassef B.G., Pape F., Poll G. (2023). Enhancing the performance of rapeseed oil lubricant for machinery component applications through hybrid blends of nanoadditives. Lubricants.

[28] Arumugam S., Sriram G., Ellappan R. (2014). Bio-lubricant-biodiesel combination of rapeseed oil: An experimental investigation on engine oil tribology, performance, and emissions of variable compression engine. Energy.

[29] Qiu S., Chen B., Yang B., Dong W., Tong Y., Li J., Xu J., Zhang L., Li C. (2023). Facile construction of graphene oxide/CeO_2_ nanohybrid for enhancing tribological properties of green rapeseed oil. Colloids and Surfaces A: Physicochemical and Engineering Aspects.

[30] Guglea D., Deleanu L., Georgescu C., Muntenia C. Tribological characteristics of rapeseed oil additivated with nano hexagonal boron nitride and graphene. Proceedings of the 22nd International Multidisciplinary Scientific GeoConference SGEM.

[31] Arumugam S., Sriram G. (2013). Preliminary Study of Nano and Microscale TiO_2_ Additives on Tribological Behavior of Chemically Modified Rapeseed Oil. Tribol. Trans..

[32] Haq I., Farooq M., Muhammd N. (2011). Some studies on the use of vegetable oils as environmentally-friendly lubricants. Tribol. Online.

[33] Singh Y., Rahim E.A., Singh N.K., Sharma A. (2023). Rapeseed oil-based biodiesel as lubricant: Frictional force and tribological analysis. Prabha Mater. Sci. Lett..

[34] Kandeva M., Kalitchin Z., Zadorozhnaya E., Vencl A. (2022). Performance characteristics of lubricant based on rapeseed oil containing different amounts of metal-containing additive. Ind. Lubr. Tribol..

[35] Bosch J., DellaCorte C. (2024). Rheological Characterization and Tribological Evaluation of Water-Based Lubricants in AISI 52100 Bearing Steel. Tribol. Lett..

[36] Tang W., Zhu X., Li Y. (2022). Tribological performance of various metal-doped carbon dots as water-based lubricant additives and their potential application as additives of poly(ethylene glycol). Friction.

[37] Wijanarko W., Khanmohammadi H., Espallargas N. (2022). Ionic Liquid Additives in Water-Based Lubricants for Bearing Steel–Effect of Electrical Conductivity and pH on Surface Chemistry, Friction and Wear. Front. Mech. Eng..

[38] Xu F., Li H., Tian B., Cui K., Dong R., Fan M., Cai M., Zhou F., Liu W. (2024). Towards superior surface behavior, tribological and mechanical response by the green, functional ionic liquid water-glycol lubricating system. Tribol. Int..

[39] Siddaraju C., Ranganatha R., Nagesh S.N., Shivukumara B., Balasubramanya H.S. (2023). Study and Comparative Analysis of Tribological Properties of Copper-Based Alloys Produced by Die Casting Method. Eng. Headw..

[40] Cihak-Bayr U., Jisa R., Franek F. (2021). Wear Protective Effects of Tribolayer Formation for Copper Based Alloys in Sliding Contacts: Alloy Dependent Sliding Surfaces and Their Effects on Wear and Friction. Tribology in Materials and Manufacturing-Wear, Friction and Lubrication.

[41] Li L., Zhuang J., Tong T., Tong J., Zhao X., Cao F., Song W., Wang D., Tian Y., Ma Y. (2023). Effect of Wet Granulation on Tribological Behaviors of Cu-Based Friction Materials. Materials.

[42] Tan Z., Guo Q., Zhai W., Zhao Z. (2012). Tribological characteristics of nickel-aluminium bronze CuAl_10_Ni_5_Fe_4_ against 30CrMnSiA steel after the prior corrosion treatment. Appl. Mech. Mater..

[43] Yaseena M.K., Mansoorb M., Ansaric H.A., Hussaind S., Khane S. (2018). Effect of Heat Treatment on Tribological Characteristics of CuAl_10_Ni_5_Fe_4_ Nickel Aluminum Bronze. Key Eng. Mater..

[44] Kazeem R.A., Fadare D.A., Ikumapayi O.M., Adediran A.A., Aliyu S.J., Akinlabi S.A., Jen T.C., Akinlabi E.T. (2022). Advances in the Application of Vegetable-Oil-Based Cutting Fluids to Sustainable Machining Operations—A Review. Lubricants.

[45] Hamnas A., Unnikrishnan G. (2023). Bio-lubricants from vegetable oils: Characterization, modifications, applications and challenges – Review. Environ. Technol. Innov..

[46] Uppar R., Dinesha P., Kumar S. (2023). A critical review on vegetable oil-based bio-lubricants: Preparation, characterization, and challenges. Environ. Dev. Sustain..

[47] (2017). Copper and Copper Alloys–Ingots and Castings.

[48] (1983). Wrought Copper Alloys; Copper-Aluminium Alloys; (Aluminium Bronze); Composition.

[49] Bernat S., Armad S., Espallargas N. (2018). Effect of Contamination on the Friction and Wear of Carboxylic Acids in Aqueous Lubricants. Tribol. Lett..

[50] Nugroho A., Mamat R., Xiaoxia J., Bo Z., Jamlos M.F., Ghazali M.F. (2023). Performance enhancement and optimization of residential air conditioning system in response to the novel FAl_2_O_3_-POE nanolubricant adoption. Heliyon.

